# Real-world evidence study on the early use of cemiplimab in the UK: REACT-CEMI (Real World evidence of advanced CSCC treatment with cemiplimab)

**DOI:** 10.3389/fimmu.2024.1408667

**Published:** 2024-07-12

**Authors:** Amarnath Challapalli, Grant Stewart, Heather Shaw, Peter John Davies, Juan Carlos Lopez-Baez, Edward C. Ottley, Stephen Kelly

**Affiliations:** ^1^ Bristol Haematology and Oncology Centre, University Hospitals Bristol and Weston NHS Foundation Trust, Bristol, United Kingdom; ^2^ Department of Clinical Oncology, Royal Cornwall Hospital, Truro, United Kingdom; ^3^ Department of Oncology, University College London Hospital and Mount Vernon Cancer Centre, Northwood, United Kingdom; ^4^ Oncology Medical Affairs Department, Sanofi-Aventis, Reading, United Kingdom; ^5^ OPEN Health, London, United Kingdom

**Keywords:** cemiplimab, CSCC, real-world, skin cancer, United Kingdom

## Abstract

**Background:**

Cemiplimab was licensed in the United Kingdom (UK) in 2019 for the treatment of patients with locally advanced and metastatic CSCC not suitable for curative surgery or radiotherapy (advanced CSCC [aCSCC]). No UK multi-center studies have investigated the real-world experience of cemiplimab post marketing authorization in aCSCC.

**Methods:**

This non-interventional retrospective study (10 UK centers) involved data collection from medical records of patients with aCSCC who initiated cemiplimab treatment between 2 July 2019 and 30 November 2020. The study period was a minimum of 12 and a maximum of 36 months post cemiplimab initiation. The primary objective was to describe the real-world clinical effectiveness of cemiplimab (primary outcome: overall response rate [ORR]).

**Results:**

Of 105 patients, 70% (n=73/105) were male (median [range] age at index of 78.5 [55.4–93.2] years); most patients (63% [n=50/80]) had an Eastern Cooperative Oncology Group (ECOG) score of 1 and 62% (n=63/102) had metastatic disease. The ORR within 12 months was 42% (95% confidence interval [CI] 32%–51%) and the disease control rate was 62% (n=65/105). The median (95% CI) real-world progression-free survival and overall survival from index was 8.6 (6.0–18.7) and 21.0 (14.7–25.2) months, respectively. The median (range) number of cemiplimab infusions was 11.0 (1.0–44.0). Eighty-seven percent experienced no cemiplimab treatment interruptions; 13% (n=14/105) interrupted treatment due to immune-related adverse reactions (irARs) (47% [n=9/19] of treatment interruption events). Eighty-five percent (n=89/105) of patients had discontinued cemiplimab treatment by the end of the study; where reasons for discontinuation were recorded, 20% (n=17/87) discontinued due to the completion of their 2-year treatment course. Nineteen percent (n=20/105) of patients experienced irARs.

**Conclusion:**

Effectiveness and safety data in this study are broadly similar to previous real-world studies of cemiplimab and the EMPOWER-CSCC1 clinical trial; with our cohort representing a broader population (included immunocompromised and transplant patients). Results support the use of cemiplimab for the treatment of aCSCC in a real-world setting.

## 1 Introduction

Patients with locally advanced cutaneous squamous cell carcinoma (laCSCC) or metastatic cutaneous squamous cell carcinoma (mCSCC) who are not candidates for curative therapies (herein described as advanced cutaneous squamous cell carcinoma [aCSCC]) have limited treatment options, with a poor prognosis and an impaired quality of life. Previous studies using chemotherapy or other systemic anti-cancer therapy have reported a median overall survival (OS) of approximately 8–15 months for this patient population (1–3).

Cemiplimab is a fully human immunoglobulin G4 (IgG4) monoclonal antibody that acts as an immune checkpoint inhibitor by binding to the programmed death-1 (PD-1) receptor, blocking the interaction between PD-1 and its ligands programmed death-ligand 1 and 2 (PD-L1 and PD-L2. Monoclonal antibodies such as cemiplimab have been shown to restore the cytotoxic capabilities of tumor antigen-specific T-cells in multiple cancers (4, 5).

Cemiplimab was granted a conditional marketing authorization by the European Commission in June 2019 as a monotherapy for the treatment of patients with aCSCC who are ineligible for curative surgery or curative radiation (6). In the National Health Service (NHS) in England, cemiplimab was initially available, pre-marketing authorization, via a Named Patient Scheme and was subsequently recommended by the National Institute for Health and Care Excellence (NICE) for reimbursement via the Cancer Drugs Fund (CDF) for advanced CSCC in July 2019 (7); followed by a recommendation for routine use in June 2022 (8). In Scotland, cemiplimab was accepted by the Scottish Medicines Consortium (SMC) in February 2020 for use on an interim basis, subject to ongoing evaluation and reassessment (9). Cemiplimab treatment is currently only reimbursed for a maximum of 2 years in England (8).

Cemiplimab is currently the first and only licensed systemic therapy for aCSCC in the UK (10, 11). Pembrolizumab, is also approved for the treatment of aCSCC in the United States (12), however it is not licensed in the UK for these patients.

The results of the EMPOWER-CSCC-1 study, a multicohort phase 2 study with 193 aCSCC patients treated with cemiplimab led to the approval of cemiplimab for aCSCC (6). The study demonstrated objective response rates of 44.9% to 50.8%, disease control rates (DCRs) ranging from 64.3% and 79.5% and a safety profile generally similar to other immune checkpoint inhibitors (12, 13). At final analysis (data cut-off: 1 March 2022), median OS had not been reached, with an estimated OS rate at 48 months of 61.8% (95% confidence interval [CI], 54.0−68.7) (14).

The generation of real-world evidence provides a broader picture of the performance of a treatment in a cohort of patients that is more reflective of routine clinical practice (15, 16).

To date, no UK multi-center studies have investigated the real-world clinical experience, effectiveness and safety outcomes of cemiplimab in aCSCC post marketing authorization. Herein, we address this evidence gap by collecting data on clinical outcomes, treatment patterns and pre-specified safety events in patients initiated on cemiplimab in routine UK clinical practice since its launch in July 2019 up to November 2020.

## 2 Study design and methods

### 2.1 Study design and setting

**Figure 1:**
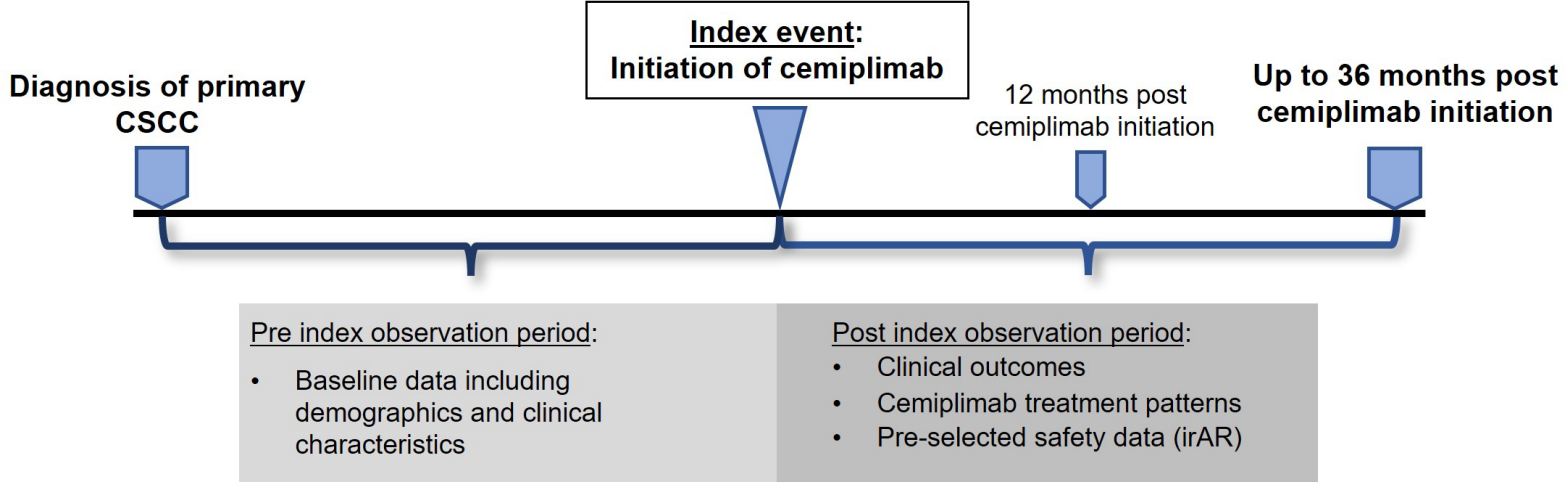
Study design and observation periods.

This was a UK, multi-center, non-interventional study with retrospective review of medical records of 105 patients with aCSCC across 10 centers. Health Research Authority (HRA) approval was obtained but no NHS Research Ethics Committee (REC) approval or patient consent was required (data collected within the NHS setting by members of the direct care team). The study design is summarized in **Figure 1**. The index event was the date of cemiplimab initiation, and the pre-index observation period was from the date of diagnosis of primary CSCC to the index date. The post-index observation period was a minimum of 12 and maximum of 36 months post-index for each patient (or until the date of death or the date of data collection if these occurred earlier than 36 months). Retrospective data collection took place between July 2022 and 31 October 2022 (follow-up of up to 36 months).

### 2.2 Patients

The population comprised adult (≥18 years) patients with aCSCC treated with cemiplimab as part of routine clinical care in the UK in participating centers. Patients treated with ≥ 1 dose of cemiplimab, initiated between 2 July 2019 and 30 November 2020, were eligible for inclusion. Patients known to have opted out of participation in any research study during the post-index observation period were excluded.

### 2.3 Study objectives and outcomes

The primary objective was to describe the real-world clinical effectiveness of cemiplimab in patients with aCSCC treated in routine clinical practice. The secondary objectives included patient demographics and clinical characteristics, treatment patterns and safety events in patients who received cemiplimab for the treatment of aCSCC as part of routine clinical practice. The primary outcome was the overall response rate (ORR) within 12 months post-initiation of cemiplimab. Secondary clinical effectiveness outcomes included: ORR within the observation period, best response, DCR, time to response (best response of CR and PR), duration of response (DoR), duration of treatment (DoT), real-world progression-free survival (rwPFS), and overall survival (OS). Safety events collected included immune-related adverse reactions (irARs) of any grade, treatment interruptions due to irARs and duration of treatment interruptions.

### 2.4 Data sources and data collection

Data were collected retrospectively from medical records by a member of the direct care team, using a bespoke pseudonymized electronic case report form (eCRF). Patient demographics and clinical characteristics were collected during the pre-index observation period; clinical outcomes, treatment patterns and pre-specified safety event data were collected during the post-index observation period. Real-world responses (i.e., partial response [PR] or complete response [CR], stable disease or progression) were taken as documented in the medical records. Where the documented response did not align with generally understood definitions of response as outlined previously (17), responses were classified by the centers investigator based on their interpretation of the information available in the medical records.

### 2.5 Statistical methods

#### 2.5.1 Data analysis

Statistical analyses were conducted using Stata V14 (StataCorp LLC) and Microsoft Excel. Data were pooled for analysis and are presented as descriptive statistics of central tendency (median; arithmetic mean or geometric mean) and dispersion (interquartile range [IQR] and/or range; standard deviation [SD] and/or 95% CI), and/or frequencies and percentages.

Responses were based on an assessment according to routine practice, as documented in medical records. The ORR within 12 months post cemiplimab initiation was analyzed as the proportion of patients who achieved either a CR or PR during this time period and presented as frequency and percentage, with two-sided 95% CI for the percentage (also provided for CR and PR). DCR represents the proportion of patients with a CR, PR or SD. OS and rwPFS were analyzed from the date of initiation of cemiplimab using the Kaplan-Meier (KM) method. For KM analysis of OS, the event was defined as death (all causes), reported as median (95% CI). Patients not recorded as having died and those lost to follow-up (LTFU) were censored on the date they were last known to be alive. For KM analysis of rwPFS, the event was defined as the date of disease progression (as first documented in medical records after initiation according to radiological progression or the date when the patient discontinued cemiplimab due to progression), or date of death (all causes). Patients alive and without disease progression, and those LTFU, were censored on the date their disease was last known not to have progressed. The time to PR, CR and best response was evaluated from the date of cemiplimab initiation until the first documentation of a PR or CR. Best response was defined as the most favorable (i.e., complete response > partial response > stable disease > progressive disease) response recorded at any point for a patient within the given time window (6 months, 12 months, 36 months). Response was also assessed in the form of a swimmer plot, according to best response during the observation period against OS (as per KM analysis), and as a Sankey plot. Patients in the following four categories were defined as immunocompromised: 1) patients previously receiving solid organ transplant or allogeneic stem cell transplant; 2) patients with significant autoimmune disease treated with immunosuppressants; 3) patients with concurrent malignancies other than CSCC (e.g., including, but not limited to, hematological malignancies); 4) patients with infection (e.g., including, but not limited to, human immunodeficiency virus [HIV], Hepatitis B and Hepatitis C).

This was a descriptive study; hence, no sample size estimation or power calculation was conducted. The target sample size of 80-100 patients was based on estimates of the precision (95% confidence limits) of the study primary outcome, based on previously published objective response rate data (12, 18).

## 3 Results

### 3.1 Patient demographics and clinical characteristics

Baseline demographics and clinical characteristics are summarized in **Table 1**. A total of 105 patients with aCSCC were included in this study. The median age at index was 78.5 years and 70% were male. Ninety percent of patients (n=94/105) were White, 89% (n=71/80) had Eastern Cooperative Oncology Group Performance Status (ECOG PS) scores of 0–1 and 11% (n=9/80) had scores ≥2. Sixty-two percent (n=65/105) of patients had no comorbidities, 26% (n=27/105) had 1 comorbidity and 12% (n=13/105) had ≥2. Overall, 17% (n=18/105) were immunocompromised and 1% (n=1/105) had a history of an organ transplant. Diabetes mellitus and chronic kidney disease (moderate to severe) were the two most common comorbidities followed by myocardial infarction and leukemia. Most patients were recorded as having either metastatic (including regional nodal and distant metastases) (62% [n=63/102]) or locally advanced (31% [n=32/102]) disease at baseline. The most common primary CSCC lesion disease sites were head/neck (69% [n=69/100]) and limbs (19% [n=19/100]). The median time from diagnosis of primary disease to diagnosis of aCSCC was 38.1 weeks (range, 0.0-487.0). Over half of the patients (55% [n=58/105]) were referred to a specialist skin cancer multidisciplinary team (SSMDT), 14% (n=15/105) were referred to a local hospital skin cancer multidisciplinary team (LSMDT) and 14% (n=15/105) to a head and neck multidisciplinary team (HNMDT). Sixty-nine percent (n=72/105) of patients had received at least one prior treatment, with 14% (n=15/105) of patients receiving more than one prior treatment. The most common prior treatment was surgery (excision or resection; 40% [n=42/105]), followed by radiotherapy (32% [34/105]).

**Table 1:** Baseline demographics and clinical characteristics.

Characteristic^1^	All patients
N^2^	105
Sex, male, n (%)	73 (70)
Age at index, median (range), (years)	78.5 (55.4–93.2)
Ethnicity	n (%)
White	94 (90)
Mixed	1 (1)
Not stated	10 (10)
ECOG PS	n (% of 80)
0	21 (26)
1	50 (63)
≥2^3^	9 (11)
Missing	25
Number of comorbidities, n (%)	
No comorbidities	65 (62)
1 comorbidity	27 (26)
≥2 comorbidities	13 (12)
Type of comorbidities at index, n (%)*	
Diabetes mellitus	14 (13)
Moderate to severe chronic kidney disease	7 (7)
Myocardial infarction	5 (5)
Leukaemia	5(5)
Cerebrovascular incident or transient ischemic attack	4 (4)
Chronic obstructive pulmonary disease	4 (4)
Congestive heart failure	4 (4)
Stage of disease at index, n (%)	n (% of 102)
Locally advanced	32 (31)
Metastatic^4^	63 (62)
Other^5^	7 (7)
Missing	3
Number of previous treatments, n (%)	
No treatment	33 (31)
One treatment	57 (54)
More than one Treatment	15 (14)
Previous treatments*, n (%)	
Surgical (excision or resection)	42 (40)
Radiotherapy	34 (32)
Chemotherapy (including platinum-based)	1 (1)
Other^6^	3 (3)
Immunocompromised patients^7^, n (%)	18 (17)
History of organ transplantation^8^, n (%)	1 (1)
Type of MDT review and referral prior to diagnosis, n (%)	
SSMDT	58 (55)
LSMDT	15 (14)
HNMDT	15 (14)
Other^9^	3 (3)
No MDT recorded	14 (13)
Primary CSCC lesion disease site	n=100
Head/neck	69 (69)
Trunk	9 (9)
Limbs	19 (19)
Genital	1 (1)
Other^10^	2 (2)
Missing	5

ECOG PS, Eastern Cooperative Oncology Group Performance Status; MDT, multidisciplinary team; LSMDT, local hospital skin cancer multidisciplinary team; HNMDT, head and neck multidisciplinary team; SSMDT, specialist skin cancer multidisciplinary team; ENT, ear, nose and throat; SD, standard deviation.

^1^At index unless otherwise stated.

^2^Denominator is N=105, unless otherwise stated.

^3^One patient considered PS 4 by the patient’s medical team due to limb amputation.

^4^Metastatic = ‘metastatic with distant metastases’, ‘metastatic with no regional node involvement’ and, ‘metastatic with regional node involvement.

^5^Other = recurrent multiple SCC (squamous cell carcinoma)’ (n=1); ‘multiple local subcutaneous metastases’ (n=1); ‘T1’ (n=1); ‘T2’ (n=3); ‘T3’ (n=1).

^6^Other = ‘Chemo-radiotherapy’ (n=1), ‘Surgery followed by adjuvant radiotherapy’ (n=2).

^7^As per the study definition; patients in the following four categories: 1) patients previously receiving solid organ transplant or allogeneic stem cell transplant; 2) patients with significant autoimmune disease treated with immunosuppressants; 3) patients with concurrent malignancies other than CSCC (e.g., including, but not limited to, haematological malignancies); 4) patients with infection (e.g., including, but not limited to, human immunodeficiency virus [HIV], Hepatitis B and Hepatitis C).

^8^For the 1 patient with a history of organ transplant recorded, this was a kidney transplant.

^9^Other = ‘ENT’ (n=1), ‘ENT – oncology and dermatology’ (n=1), ‘Neuro-oncology’ (n=1).

^10^Other = ‘skin’ (n=2).

*Not mutually exclusive.

### 3.2 Real-world effectiveness of cemiplimab

**Figure 2:**
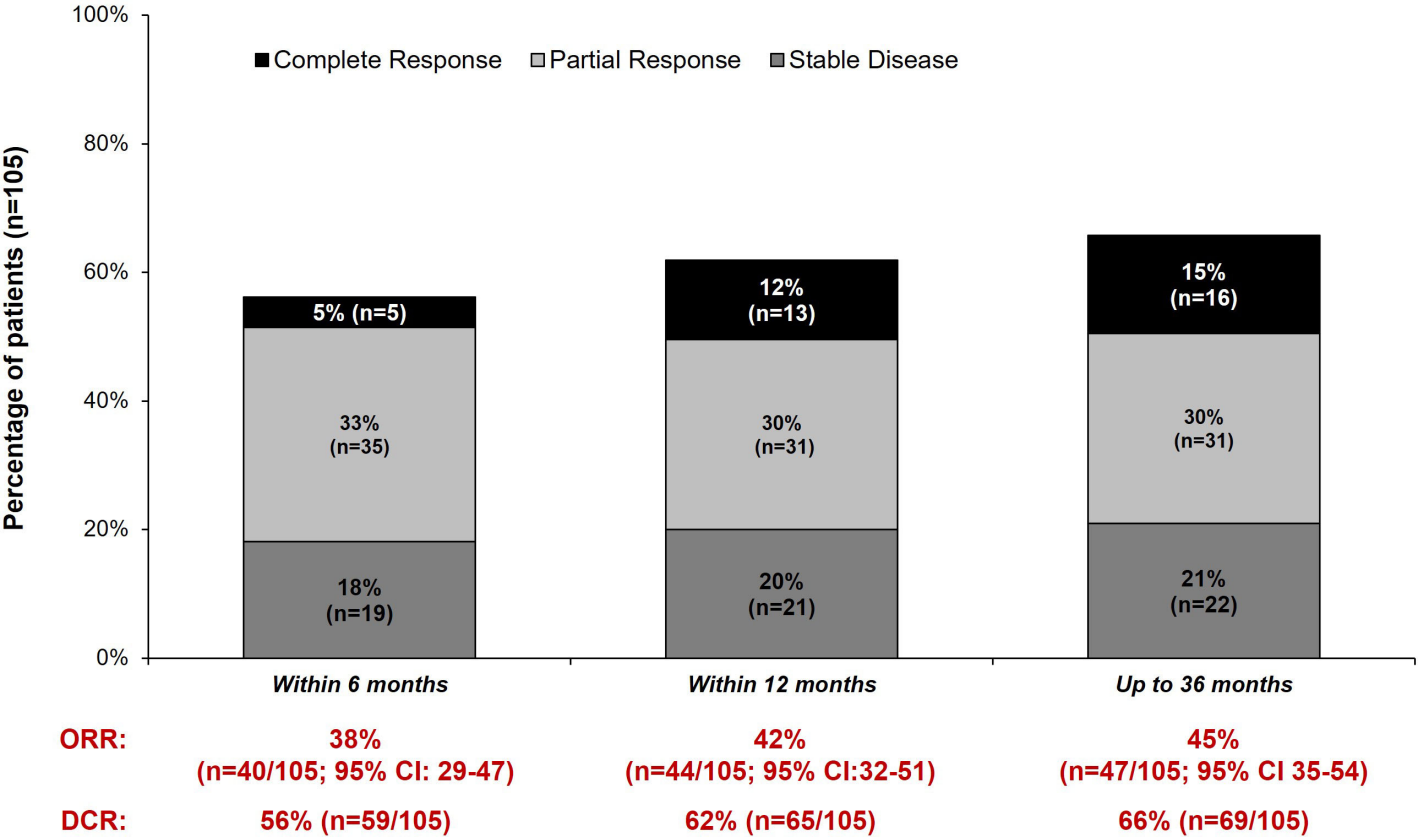
Real-world ORR, best response and DCR within 6-, 12- and 36 months post-index. Real-world best response within 6- 12- and 36 months post-index is shown. In addition to a complete response, partial response or stable disease, additional categories were also present: The number of patients with progressive disease (6 months: 12% [n=13/105]; 12 months: 14% [n=15/105]; 36 months: 16% [n=17/105]), inconclusive responses (6 months: 1% [n=1/105]; 12 months: 1% [n=1/105]; 36 months: 3% [n=3/105]), no documented response within the time window (6 months: 15% [n=16/105]; 12 months: 8% [n=8/105]; 36 months: 0% [n=0/105]), Died or discontinued treatment before 12 weeks (i.e., prior to first response scan) without a recorded response (6 months: 14% [n=15/105]; 12 months: 14% [n=15/105]; 36 months: 14% [n=15/105]) and other (1% [n=1/105] for all timepoints). One patient stopped treatment 6 months post-initiation without a recorded treatment response. The disease control rate (DCR) and overall response rate (ORR) within 6-, 12- and 36 months is also shown.

**Figure 3:**
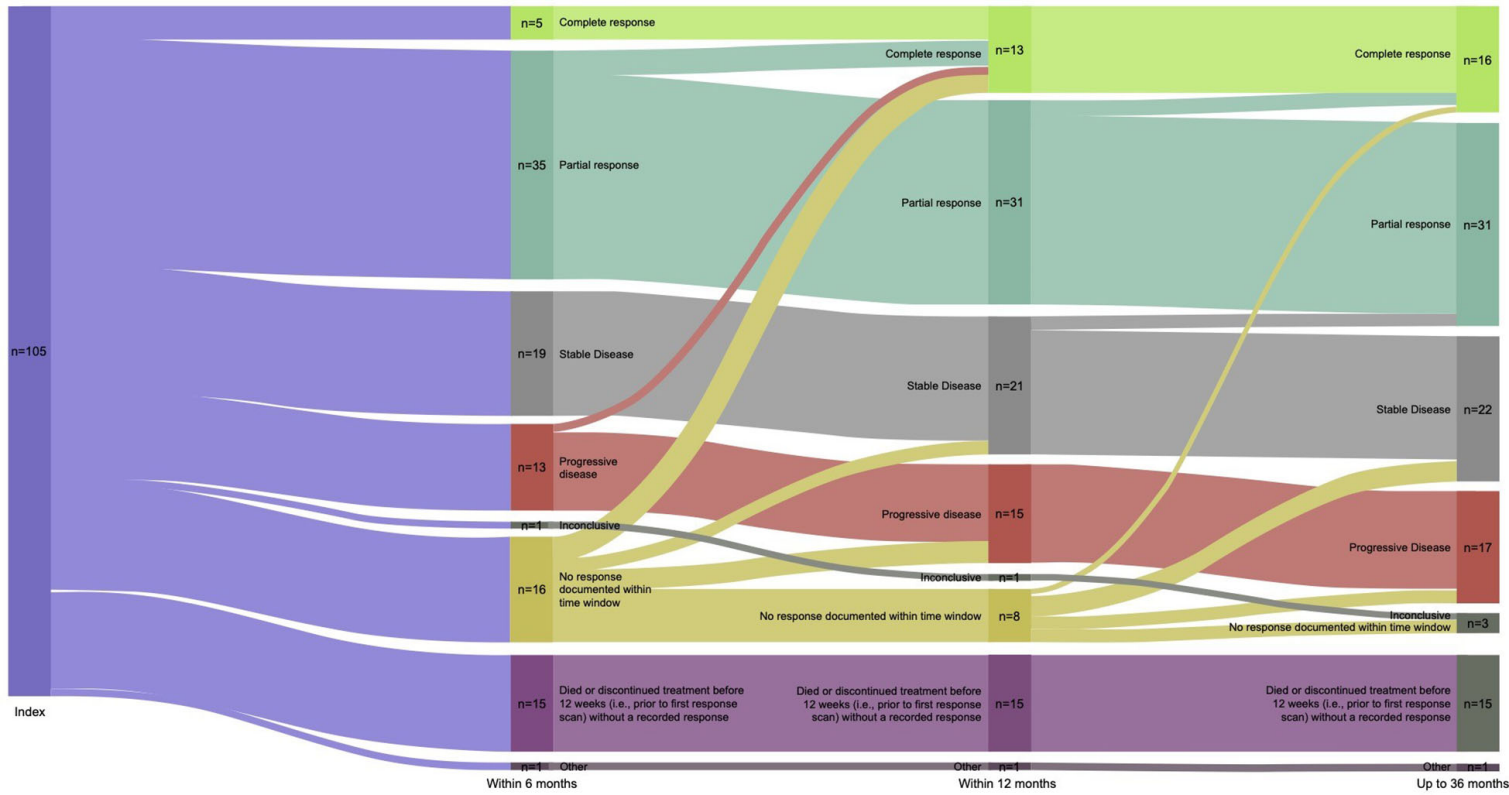
Sankey plot of response to cemiplimab. A Sankey plot of the best response to cemiplimab within 6-, 12- and 36 months is shown, depicting change in best response for patients at each timepoint. This is the best recorded response leading up to each timepoint, patients cannot be categorized into a less favorable response category.

**Figure 4:**
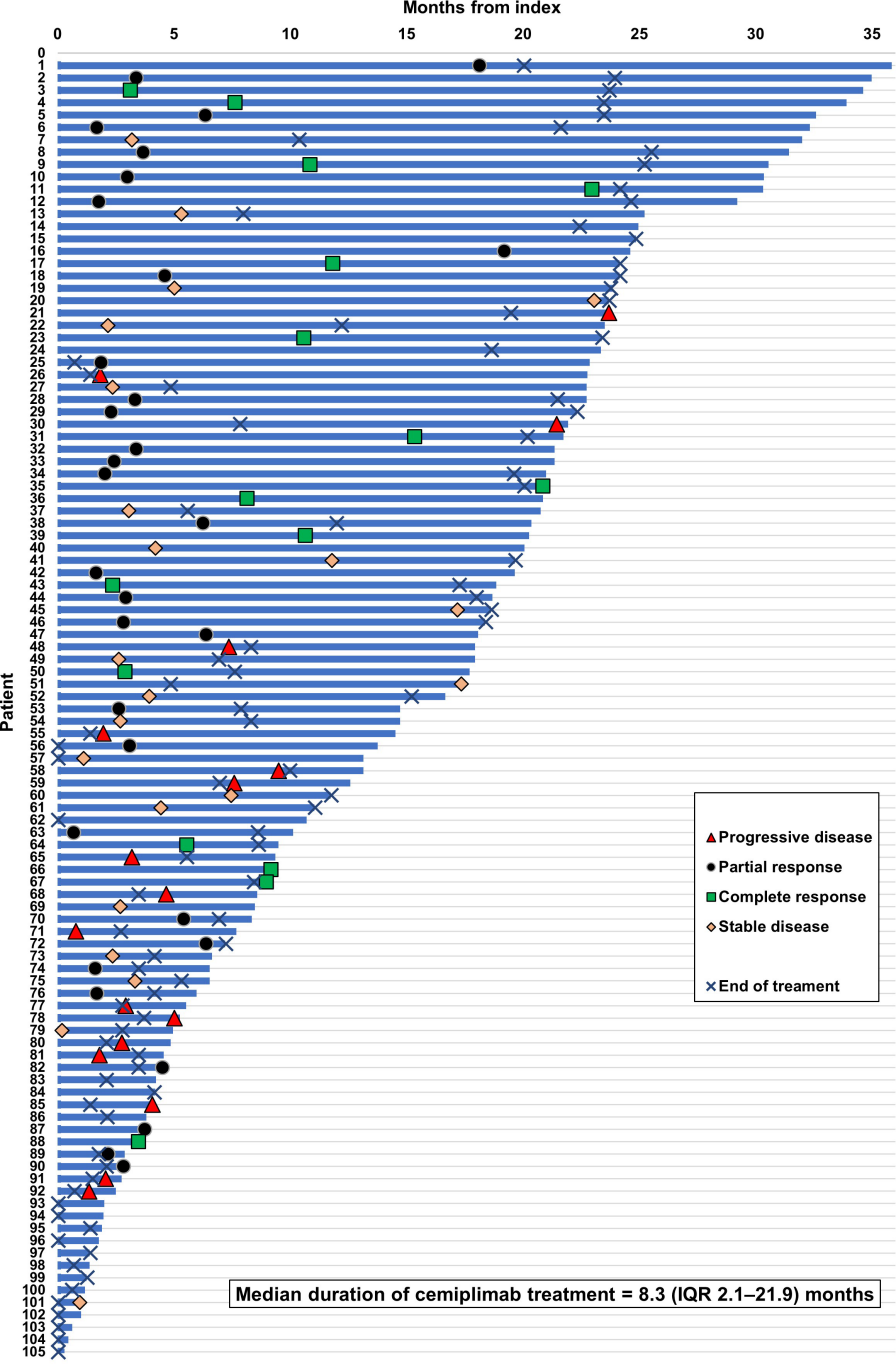
Swimmer plot of response to cemiplimab. Swimmer plot shows OS (calculated as time until death, LTFU or last contact within observation), with treatment responses of CR, PR or PD plotted according to when they occurred during the observation period. The median duration of treatment was 8.3 (IQR 2.1–21.9) months.

**Figure 5:**
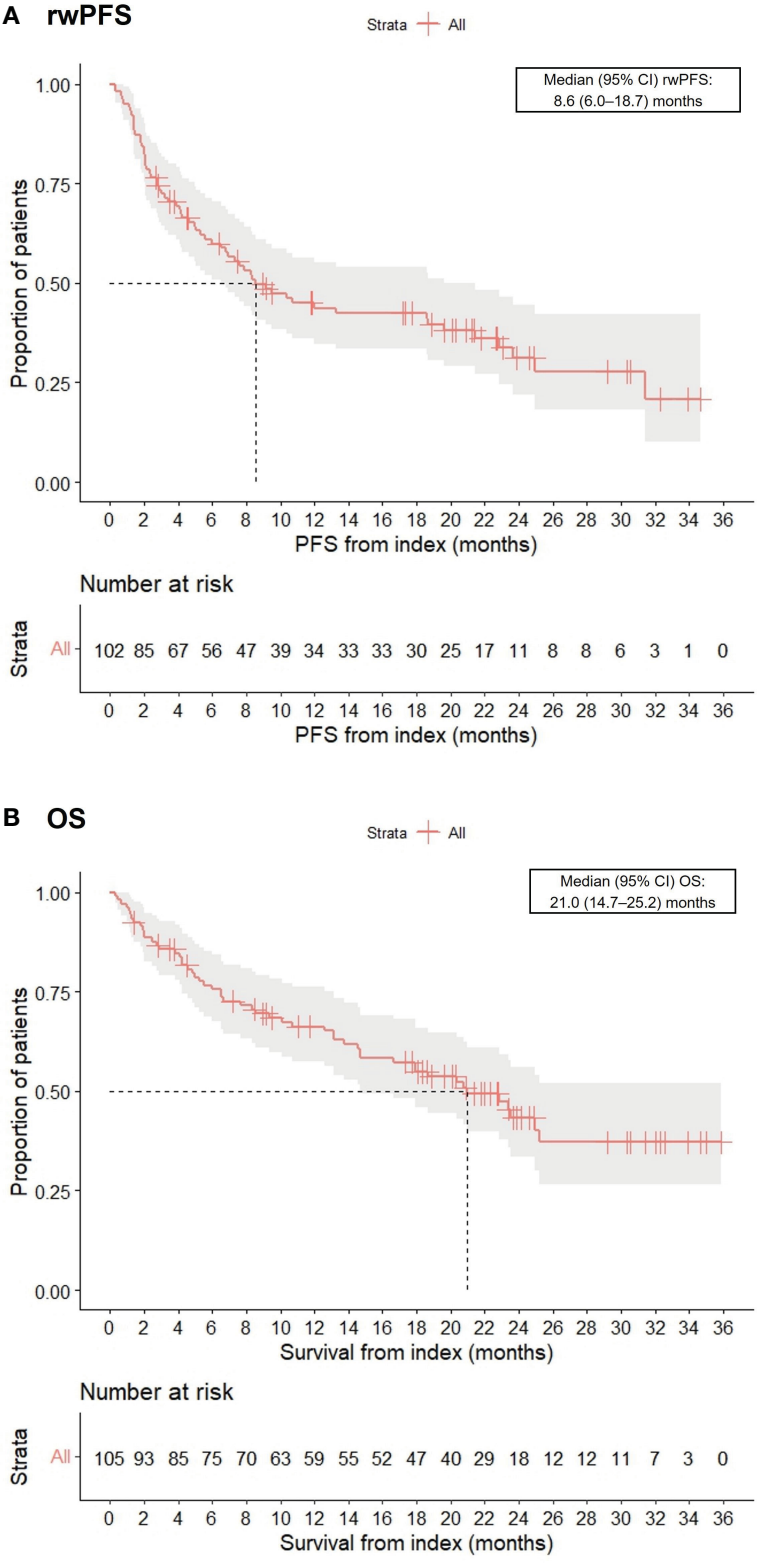
Kaplan-Meier charts of real-world progression-free survival (rwPFS) **(A)** and overall survival (OS) **(B)**.

Real-world best response rates increased numerically over time, with ORR within 6-, 12- and up to 36-months post-initiation of cemiplimab being 38% (95% CI, 29%–47%; n=40/105), 42% (95% CI, 32%–51%; n=44/105), and 45% (95% CI, 35%–54%; n=47/105), respectively (**Figure 2**. This included a numerical increase in CR rate as best response from 5% (n=5/105) to 12% (n=13/105) between 6 months and 12 months (**Figure 2**). Best response to cemiplimab is also summarized in a Sankey plot (**Figure 3**) and in a Swimmer plot (**Figure 4**). By the end of the observation period, most patients had a documented assessment of treatment response, leading to a numerical increase in both the ORR (42% vs. 45%) and the proportion of patients achieving a CR (12% vs 15%) between 12 months and the end of the observation period (up to 36 months). A numerical increase in the proportion of patients with SD (20% [n=21/105] vs 21% [n=22/105]) and PD (14% [n=15/105] vs. 16% [n=17/105]) was also observed. A DCR of 56% (n=59/105), 62% (n=65/105) and 66% (n=69/105) was achieved within 6-, 12- and 36-months post-index, respectively (**Figure 2**). The median time to PR was 3.0 (IQR, 2.1–4.6) months and to CR was 9.1 (IQR, 5.0–11.1) months and the median time to a best response (CR or PR) was 3.5 (IQR, 2.5–7.9) months (**Table 2**). The median DoT was 8.3 (IQR, 2.1–21.9) months, however, for responding patients, long DoR were observed with a median DoR of 21.2 (IQR: 15.2–27.4) months (**Table 2**). The median rwPFS observed was 8.6 (95% CI, 6.0–18.7) months (**Figure 5A**) with a median OS of 21.0 (95% CI, 14.7–25.2) months for the study population (**Figure 5B**). Additional rwPFS, OS and ORR rates stratified by immunocompromised status are included in the supplementary information (**Supplementary Tables S3**–**S5**, **Supplementary Figures S1**, **S2**).

**Table 2:** Response times and duration of cemiplimab treatment.

Median response times and duration of treatment	n	months (IQR)
Duration of response within observation period^1^	47	21.2 (15.2-27.4)
Time to best response	47	3.5 (2.5-7.9)
Time to PR^2^	31	3.0 (2.1-4.6)
Time to CR	16	9.1 (5.0-11.1)
Duration of treatment within observation period^3^	105	8.3 (2.1-21.9)

CR, complete response; IQR, interquartile range; PR, partial response.

^1^Duration of response: The time from the first documentation of a CR or PR to cemiplimab in medical records until first documentation of disease progression or death. If patient did not die or experience disease progression during the post-index observation period and the data collection date was less than 36 months from index, the difference between data collection date and first response date was taken as the response duration.

^2^Time (months) to a best response of ‘partial response’.

^3^Duration of treatment: The time from cemiplimab initiation to the documented date of treatment discontinuation. DoT was calculated from the time of cemiplimab initiation to the documented date of discontinuation. For patients without discontinuation during the observation period, duration of cemiplimab treatment was calculated from the time of cemiplimab initiation until the date of data collection (where the time between date of data collection and date of cemiplimab initiation was < 36 months); otherwise, 36 months post-initiation of cemiplimab initiation was used.

### 3.3 Cemiplimab treatment patterns

Cemiplimab treatment patterns are summarized in **Table 3**. The median (range) number of cemiplimab infusions during the observation period was 11.0 (1.0–44.0). A total of 13% (n=14/105) of patients experienced cemiplimab treatment interruptions, which included 47% (n=9/19) of treatment interruption events that were due to an irAR. Other interruptions included patient decision and other adverse reaction (non-immune related) in 16% (n=3/19) respectively. A total of 85% (n=89/105) of patients discontinued cemiplimab treatment (any reason). For patients with discontinuation data available (n=87), 34% (n=29/87) discontinued due to disease progression and 20% (n=17/87) discontinued due to the completion of their 2-year treatment course. Within the study period, 12% (n=10/87) of patients were recorded to have discontinued due to death (any cause). Additional information on irARs and cemiplimab treatment interruptions in immunocompromised patients can be found in the supplementary information (**Supplementary Tables S6**, **S7**).

**Table 3:** Cemiplimab treatment patterns.

	n	n=105
**Number of infusions, median (range)**	105	11.0 (1.0-44.0)
**Cemiplimab treatment interruptions**	105	% (of 105)
Treatment interrupted	14	13
No interruption	91	87
**Number of treatment interruptions**	105	% (of 105)
1 interruption	11	10
>1 interruption	3	3
**Reasons for cemiplimab treatment interruption**	19	% (of 19)
irAR	9	47
Patient decision	3	16
Other adverse reactions (non-immune-related)	3	16
Infection	2	11
Surgery	1	5
Other^1^	1	5
**Treatment discontinuation**	105	% (of 105)
Treatment discontinued^2^ (any reason)	89	85
No discontinuation experienced	16	15
**Reasons for discontinuation**		% (of 87)
Treatment course completed	17	20%
Disease progression	29	34%
irAR	15	17%
Death (any cause)	10	12%
Patient decision	5	6%
Other adverse reactions (non-immune related)	3	3%
Other^3^	8	5%
Missing	2	–

irAR, immune-related adverse reaction.

^1^Other = ‘given a 6 week break to avoid exposure to healthcare environments during the COVID-19 outbreak’ (n=1).

^2^Includes permanent discontinuations (n=88) and temporary discontinuations (n=4).

^3^Other = Includes atrial flutter (n=1), infection (n=1), toxicity (n=1), hospitalisation (n=1), patient with clinical deterioration/decline in health (n=3), patient quality of life/frailty of age (n=1).

### 3.4 Real-world cemiplimab safety

A total of 19% (n=20/105) of patients experienced irARs of any grade during the post-index observation period (**Table 4**). Additionally, 6% (n=6/105) of patients (with a total of 9 interruption events) also had treatment interruptions due to irARs. The median (range) duration of interruption was 22 days (21-57). A summary of the management of irARs is summarized in **Supplementary Table S1** and **Supplementary Table S2**.

**Table 4:** Real-world safety of cemiplimab.

	n	% (of 105)
irAR (of any grade) experienced during the post-index observation period	105	
Patients with irARs	20	19%
Patients with no irARs	85	81%
Interruptions due to irARs	105	
Experienced treatment interruptions	6	6
No treatment interruptions	99	94

## 4 Discussion

To our knowledge, REACT-CEMI is the first multi-center UK real-world study to investigate the early clinical experience, effectiveness and safety of cemiplimab for the treatment of aCSCC post marketing authorization. The results provide important insights into UK practice and they outline the early management of aCSCC and the clinical characteristics of these patients. Furthermore, results provide a real-world outlook on the early outcomes of aCSCC patients who received cemiplimab within the first 2 years post marketing authorization, and patients who initiated cemiplimab during the coronavirus disease-2019 (COVID-19) pandemic.

The primary objective of the study was to describe the real-world clinical effectiveness of cemiplimab in patients with advanced CSCC treated in routine clinical practice. As part of this, ORR within 12 months of cemiplimab initiation, was assessed as the primary outcome of the study, with 42% of patients responding to treatment at 12 months. This percentage relatively increased to 45% over the whole study period which was driven by a numerical increase in both CR and PR rates over time as patient responses deepened (**Figure 3**). These responses were marginally higher than those reported for cemiplimab in the UK early access programme (19), and in line with previously reported responses as part of a French and Italian early access schemes (20, 21). More recent real-world studies have investigated the use of cemiplimab and pembrolizumab in aCSCC with higher response rates reported (22, 23). The phase 2 EMPOWER-CSCC-1 trial reported objective response rates of 46.4% to 50.8%, which are broadly similar to the ORRs observed in this study within 12- and 36-months post cemiplimab initiation. In addition, the DoR reported in the current study was shorter than that reported in the EMPOWER-CSCC-1 trial. This could be attributed to the shorter follow-up period and the real-world setting of the current study.

The overall DCR (up to 36 months) in our study (66%) was also broadly similar to the DCRs reported in the Italian study and the NPS (21). However, the follow-up for the Italian study was shorter, at 9 months. Our results demonstrated a median rwPFS of 8.6 months and a median OS of 21 months. These compare favorably against the UK and French early access studies (19, 20) but are shorter than the median PFS and OS reported in the EMPOWER-CSCC1 trial and the recent Australian real-world evidence study (23). The reasons for this could be that, firstly, our data are reflective of early clinical experience with potentially immature treatment and referral pathways. In addition, the COVID-19 pandemic was ongoing during the observation period, therefore we cannot rule out that this may have had an impact on delaying or commencing treatment. Indeed, a range of studies have highlighted the negative impact of COVID-19 on CSCC treatment accessibility and diagnosis (24, 25). For instance, a retrospective Serbian study demonstrated that CSCC patients post-pandemic exhibited statistically significant increases in the largest tumor diameter and had an increased rate of invasive disease (25). Further to this, an Italian study reported that the reduced level of access to medical care during the COVID-19 pandemic led to a documented diagnostic delay (24).

The current study represents a more diverse patient population, in terms of age, comorbidities, ECOG score and immunocompromized status. Some of these patients, such as patients with immunocompromized status or with transplant history (representing 18% of patients in the present study) or with certain comorbidities, would have been excluded from the EMPOWER study. In terms of the patient demographics and clinical characteristics, based on the age of the patients included in the study, it was evident that this was an elderly cohort; consistent with other real-world studies and the natural history of CSCC generally (26).

Progression of CSCC is often fast, hence, a timely referral plays a crucial role in outcomes for patients (19). Whilst most patients (87%) were referred via an MDT prior to diagnosis, 13% of patients had no MDT recorded. Furthermore, while most patients were seen at an SSMDT (55%), approximately 1 in 8 patients were seen by other MDTs.

Overall, cemiplimab appeared to be well tolerated; eighty-seven percent of patients had no treatment interruptions and a total of 19% (n=20/105) experienced irARs. In 17% of patients (n=15/87), irARs led to cemiplimab treatment discontinuation. This is broadly consistent with the rate of irARs seen in other retrospective real-world studies of cemiplimab in aCSCC (27).

Limitations of this study included the retrospective study design based on secondary use of data. As such, the results are dependent on the completeness and quality of the medical records and the reliability of the abstraction of data from them. From a statistical perspective, this was a descriptive study, hence, no analyses to control for confounding were conducted. In addition, response/progression assessments were real-world estimates and were impacted by the frequency by which assessments were made in routine clinical practice. Where real-world response/progression outcomes were not clearly documented in the medical records, these were retrospectively classified by the center investigator based on the available information, where possible, which could have led to potential bias in the absence of blinded centralized review.

The results are expected to be broadly generalizable to the wider source population. Firstly, in relation to the study center selection, centers were selected based on post-marketing authorisation use of cemiplimab which included centers with early experience with cemiplimab across England, Wales and Scotland. Due to this, results obtained are likely to be representative of early use of cemiplimab and representative of UK practice. As cemiplimab reimbursement is restricted to 2 years in the UK, experience of this treatment in the UK may differ from other healthcare settings with longer reimbursement.

This study is the first description of the early real-world clinical experience of cemiplimab for the treatment of aCSCC in a multi-center UK clinical setting. The ORR observed in the present study was consistent with other real-world studies of cemiplimab conducted globally, including the Named Patient Scheme conducted in the UK and the EMPOWER-CSCC1 trial. The results of this study support the use of cemiplimab for the treatment of aCSCC in a real-world setting and will help inform clinical decisions.

## Data availability statement

The datasets presented in this article are not readily available because the original contributions presented in the study are included in the article/**Supplementary Material**. Further inquiries can be directed to the corresponding author. Requests to access the datasets should be directed to amarnath.challapalli@uhbw.nhs.uk.

## Ethics statement

The requirement of ethical approval was waived by Health Research Authority (HRA) for the studies involving humans, because this is not required for a non-interventional study with retrospective review of medical records. The studies were conducted in accordance with the local legislation and institutional requirements. The ethics committee/institutional review board also waived the requirement of written informed consent for participation from the participants or the participants’ legal guardians/next of kin because this was a non-interventional study with retrospective review of medical records.

## Author contributions

AC: Conceptualization, Writing – original draft, Writing – review & editing. GS: Writing – original draft, Writing – review & editing. HS: Writing – original draft, Writing – review & editing. PD: Conceptualization, Writing – original draft, Writing – review & editing. JL-B: Conceptualization, Writing – original draft, Writing – review & editing. EO: Writing – original draft, Writing – review & editing. SK: Conceptualization, Methodology, Writing – original draft, Writing – review & editing.
