# Supplementary material for: Real-world evidence study on the early use of cemiplimab in the UK: REACT-CEMI (Real World evidence of advanced CSCC treatment with cemiplimab)
Source: Front Immunol. 2024 Jul 12;15:1408667. doi: 10.3389/fimmu.2024.1408667 (PMC11272471; doi:10.3389/fimmu.2024.1408667)

Supplementary Material

# Supplementary Data

**Table S1: Proportion of patients treated with anti-inflammatory drugs for irARs overall and by type**

| **Patients treated with or without anti-inflammatory drugs for irARs** | **n** | **% (n=20)** |
| --- | --- | --- |
| Without anti-inflammatory drugs | **1** | 5% |
| With anti-inflammatory drugs | **19** | 95% |
| **Type of anti-inflammatory drug^1^** | **n (events)** | **% (n=30^2^)** |
| High-dose prednisolone | 19 | 63% |
| Low-dose prednisolone | 2 | 7% |
| Methylprednisolone | 2 | 7% |
| Mycophenolate | 2 | 7% |
| Infliximab | 1 | 3% |
| Hydrocortisone | 1 | 3% |
| Clobetasol | 1 | 3% |
| Triamcinolone acetonide | 1 | 3% |
| Vedolizumab | 1 | 3% |
| Missing | 1 | - |
| ^1^Data are not mutually exclusive.  ^2^30 prescriptions for anti-inflammatory drugs across the 19 patients. |  |  |

**Table S2: Initial dose of anti-inflammatory drug used to treat irARs at onset of irAR and for the duration of irARs by steroid type**

| **Dose (mg/kg/day)** | **High-dose prednisolone** | |
| --- | --- | --- |
|  | **n** | **% (n=15)** |
| 0.5 mg/kg/day | 1 | 7% |
| 1.0 mg/kg/day | 11 | 73% |
| 2.0 mg/kg/day | 3 | 20% |
| Total (non-missing) | 15 | - |
| Missing | 4 | - |
| **Dose (mg/kg/day)** | **Low-dose prednisolone** | |
|  | **n** | **% (n=2)** |
| 0.3 mg/kg/day | 1 | 50% |
| 0.5 mg/kg/day | 1 | 50% |
| Total (non-missing) | 2 | - |
| Missing | 0 | - |
| **Dose (mg/kg/day)** | **Methylprednisolone** | |
|  | **n** | **% (n=2)** |
| 1.5 mg/kg/day | 1 | 50% |
| 2 mg/kg/day | 1 | 50% |
| Total (non-missing) | 2 | - |
| Missing | 0 | - |
| **Dose (mg/kg/day)** | **Mycophenolate** | |
|  | **n** | **% (n=0)** |
| Total (non-missing) | - | - |
| Missing | 2 | - |
| **Dose (mg/kg/day)** | **Infliximab** | |
|  | **n** | **% (n=1)** |
| 3 mg/kg/day | 1 | 100% |
| Total (non-missing) | 1 | - |

**Table S3: Real-world progression-free survival, overall survival, and overall response rate by immunocompromized status**

| **Outcome** | **Immunocompromized** | **Non-immunocompromized** |
| --- | --- | --- |
|  | **N=18** | **N=84** |
| **rwPFS (95% CI)**  12 months  24 months  Median (months) | 19.1% (7.0%—51.7%)  19.1% (7.0%—51.7%)  4.0 (2.8—NE) | 49.1% (39.1%—61.8%)  32.6% (21.8%—48.9%)  12.0 (7.6—23.7) |
|  | **N=18** | **N=87** |
| **OS (95% CI)**  12 months  24 months  Median (months) | 76.2% (58.2%—99.7%)  33.5% (16.0%—70.4%)  22.9 (20.4—NE) | 64.2% (54.7%—75.4%)  48.4% (38.3%—61.1%)  21.0 (14.5—NE) |
|  | **N=18** | **N=87** |
| **ORR, n (%)**  Achieved  Not achieved | 10 (56%; 95% CI, 32.6%—78.5%)  8 (44%) | 34 (39%; 95% CI, 28.8%—49.3%)  53 (61%) |
| Key: CI – confidence interval, NE – not estimable, OS – overall survival, rwPFS – real-world progression-free survival. | | |

**Table S4: Overall response rate (ORR) in immunocompromized patients by time**

| **Immunocompromized patients’ overall response rate** | **ORR within 12 months** | | **ORR during post initiation period** | |
| --- | --- | --- | --- | --- |
|  | **n** | **% (n=18)** | **n** | **% (n=18)** |
| **Achieved** | **10** | 56% | **10** | 56% |
| Complete Response (CR) | 2 | 11% | 2 | 11% |
| Partial Response (CR) | 8 | 44% | 8 | 44% |
| **Not achieved** | **8** | 44% | **8** | 44% |
| Stable Disease | 3 | 17% | 4 | 22% |
| Progressive Disease | 1 | 6% | 1 | 6% |
| Response Inconclusive | 0 | 0% | 0 | 0% |
| No response documented within time window | 1 | 6% | 0 |  |
| Died or discontinued treatment without recorded response within 10 weeks | 2 | 11% | 2 | 11% |
| Other* | 1 | 6% | 1 | 6% |

**Table S5: Overall response rate (ORR) in non-immunocompromized patients by time**

| **Non-immunocompromized patients’ overall response rate** | **ORR within 12 months** | | **ORR during post initiation period** | |
| --- | --- | --- | --- | --- |
|  | **n** | **% (n=87)** | **n** | **% (n=87)** |
| **Achieved** | **34** | 39% | **37** | 43% |
| Complete Response (CR) | 11 | 13% | 14 | 16% |
| Partial Response (CR) | 23 | 26% | 23 | 26% |
| **Not achieved** | **53** | 61% | **50** | 57% |
| Stable Disease | 18 | 21% | 18 | 21% |
| Progressive Disease | 14 | 16% | 16 | 18% |
| Response Inconclusive | 1 | 1% | 3 | 3% |
| No response documented within time window | 7 | 8% | 0 |  |
| Died or discontinued treatment without recorded response within 10 weeks | 13 | 15% | 13 | 15% |
| Other* | 0 | 0% | 0 | 0% |

**Table S6: Proportion of immunocompromized patients experiencing immune-related adverse reactions (irARs) of any grade (where reported in notes) [At Initiation]**

|  | **n** | **% (n=18)** |
| --- | --- | --- |
| Adverse reactions - any grade | 5 | 28% |
| No adverse reactions | 13 | 72% |

**Table S7: Proportion of patients with cemiplimab treatment interruptions due to experiencing immune-related adverse reactions (irARs)**

|  | **n** | **% (n=18)** |
| --- | --- | --- |
| Experienced treatment interruptions | 3 | 17% |
| Experienced no treatment interruptions | 15 | 83% |

**Figure S1. Kaplan-Meier Chart of overall survival stratified by immunocompromised status**


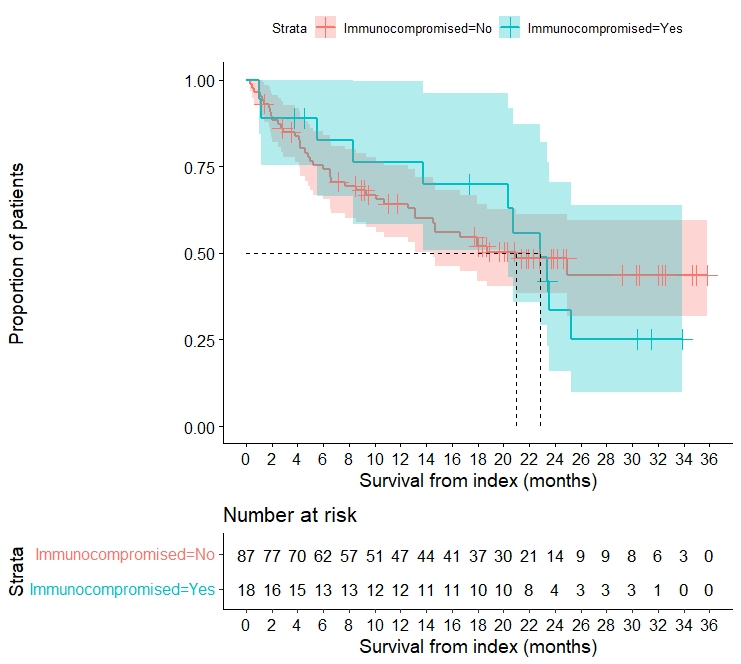


**Figure S2. Kaplan-Meier Chart of progression-free survival stratified by immunocompromised status**


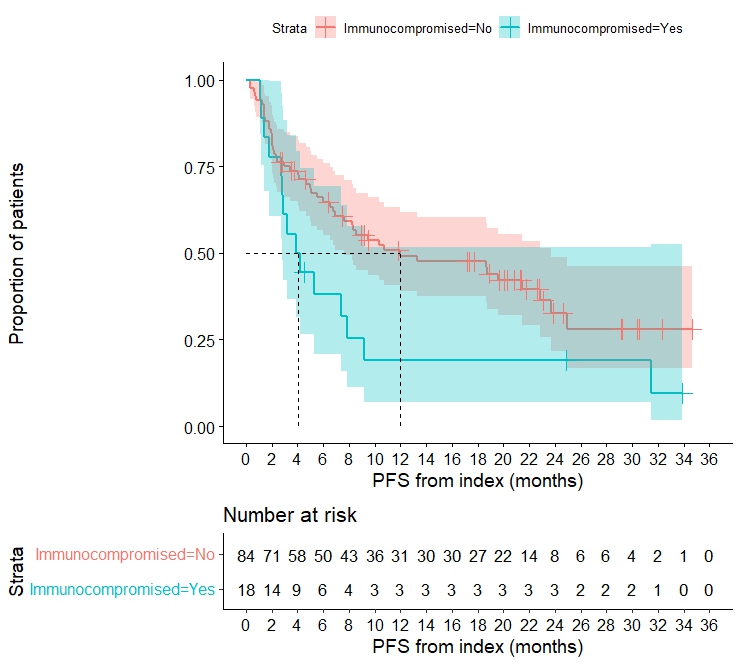

Supplement: Supplementary file 1 [file DataSheet_1.docx]
